# Phycocyanin content and nutritional profile of *Arthrospira platensis* from Mexico: efficient extraction process and stability evaluation of phycocyanin

**DOI:** 10.1186/s13065-021-00746-1

**Published:** 2021-04-05

**Authors:** Sanghamitra Khandual, Edgar Omar Lopez Sanchez, Hugo Espinosa Andrews, Jose Daniel Padilla de la Rosa

**Affiliations:** Centro de Investigación Y Asistencia en Tecnología Y Diseño del Estado de Jalisco. Av. Normalistas 800 Colinas de La Normal, C.P. 4227 Guadalajara, Jalisco Mexico

**Keywords:** Phycocyanin, Stability, Natural colorant, Extraction, Purification

## Abstract

Phycocyanin is a blue natural food colorant with multiple health benefits. Here we propose an efficient phycocyanin extraction method from *Arthrospira platensis* from Mexico. Three extraction methods were applied to optimize the extraction process, using water and buffer as solvents, with three pH values at two agitation times. The highest phycocyanin, 54.65 mg/g, was extracted from dry biomass with water as a solvent using an ultrasonication bar. The optimum condition of extraction was determined to be 1:50 biomass/solvent ratio for dry biomass, with the freeze/thaw method for 20 min repeated twice, and then agitated at 120 rpm for 24 h. The phycocyanin content was 48.88 mg/g biomass, with a purity of 0.47. For scalable phycocyanin productivity, the sonication method is recommended as there is no statistical difference. The phycocyanin stability was best at − 20 °C storage temperature at pH 7 for 35 days. Partial purification with ammonium sulfate was found to be suitable as a fractional precipitation method, first at 0–20% and then 20–65%, to get purity nearly 1. Total protein was found to be 55.52%, and total amino acids after phycocyanin extraction was 33%. The maximum phycocyanin yield using water as a solvent was the most interesting result regardless of the method used for extraction.

## Introduction

Phycocyanin is a pigment-protein complex in the light-harvesting phycobiliprotein family, present along with allophycocyanin and phycoerythrin [1]. It is a light blue color pigment, and absorbs orange and red light at 620 nm and emits fluorescence at approximately 650 nm. The people of Mexico harvest Arthrospira for nourishment [2]. *Arthrospira platensis* (*spirulina)* draws attention because of its nutritional and medicinal properties [3]. Phycocyanin (C-PC) is a water-soluble compound found in cyanobacteria and in up to 15% of proteins in *S. platensis* [4]. Phycocyanin has antioxidant, anticancer, and anti-inflammatory properties [5]. According to; Cuellar-Bermudez et al. [6] and Ravi et al. [7], polysaccharides and phycobiliproteins from certain algal species can inhibit the growth of tumor cells. Phycocyanin extracted from *S. platensis* showed health benefits, such as improved immune function, regeneration of zooblasts, and inhibition of cancer cell growth [8–10]. So it is vital to include phycocyanin as a functional food ingredient to fight or prevent cancer and as an antioxidant-enriched supplement. Recently it was reported that a peptide of *Arthrospira platensis* reduced blood pressure levels through a PI3K/AKT/eNOS-dependent mechanism [11].

Phycocyanin is a blue natural food colorant that can be used as a functional food ingredient for several health benefits and as a natural colorant. For dairy products, it can be used in additives such as dyes with stabilizers and emulsifiers during the manufacturing process. The biosecurity of food is threatened due to the addition of synthetic additives and artificial colors in food, as they sometimes inhibit the absorption of some nutrients and may cause allergic reactions. Sometimes synthetic additives impart an undesirable flavor and color and can destroy vitamins in food. The use of sustainable and eco-friendly chemicals as colorants is very limited due to the lack of alternatives and few color shades [12]. The overutilization of a few known natural plant extracts as colorants has threatened the ecosystem’s sustainability [13, 14]. Hence, there is a need to search for alternative sources of natural dyes that are renewable and do not affect the ecosystem balance [15, 16]. Due to the high growth rate, many color shades, and widespread occurrence of algae in different climatic conditions, they may be the best sources of natural dyes [17, 18] for industrial applications.

Due to several drawbacks there is increasing awareness of artificial colors, and the trend is to incorporate natural ingredients into functional foods that are safe, with additional functional value to improve the quality of the food. Several value-added bioactive compounds have shown good acceptance in the market. Phycocyanin from *Arthrospira* (Spirulina) can be used to enrich the nutritional value and also give a natural color to dairy products. There is a wide range of food formulations with cyanobacteria biomass. It has been widely used as an additive or supplement to cereals or salads. Arthrospira is effectively used as a natural and slimming food in the USA and Europe. *Arthrospira* concentrates can be used in the preparation of new foods [19]. Seventy percent of the current Arthrospira market is for human consumption as healthy food [20]. Several studies found food fortified with whole Arthrospira biomass, except one report with isolated C-PC [21]. Arthrospira was incorporated as a functional ingredient into pasta [22–24], biscuits and cookies [25–27], extruded products [28–30], yogurt and acidophilus milk [31, 32], baby food formulas [33], and bread [34]. However, the thermal processing of these food products leads to the destruction of C-PC, resulting in a green-yellowish color due to partially retained carotenoids and chlorophyll. This was ignored in almost all studies, and few reports demonstrate C-PC degradation at higher temperatures [35]. In Mexico, there are very few reports on native *Arthrospira* species and their bioactive compounds. *S. maxima* is used as a supplement for cookies as part of the national breakfast program for children [36].

Rout et al. 2015 [37], reported on three *A. platensis* strains from Mexico; in spite of their morphological variations, they were grouped genetically into one cluster by molecular identification. *A. platensis* NPS-1Tex strain used in this work is one of them. Due to its easy cultivation for biomass productivity, it is popular in Mexico and is in production by the Biomex company. So it is important to optimize this strain for suitable industrial-scale phycocyanin and protein production. Despite the high content of phycocyanin in cyanobacteria (60–70 mg/g), commercial productivity is limited because it depends significantly on biomass quality and culture conditions [38]. Again, the extraction method is the key factor for optimum yield and commercialization of phycobiliproteins [39]. The extraction of phycobiliproteins involves cell rupture methodology to release these proteins with a good solvent that can be further used in the food sector without toxicity and at lower cost. Recently, mechanical cell rupture methods have been popular for large-scale productivity productions [40, 41] with high product yields. Furthermore, there is a risk of contamination or degradation in achieving the hygienic standards required for application in food and pharmaceutical products. Including *Arthrospira e*xtract or partially purified food-grade phycocyanin as a functional food ingredient and a natural food color still lacks technological development. A better understanding of the stability conditions in the food matrix is needed [42, 43].

Here we tried to optimize efficient extraction and a partial purification methodology and evaluate better conditions for phycocyanin stability for the crude extract from a native *Arthrospira platensis* species, which can be used in the future for food matrixes in industrial sectors to enhance food quality and color.

## Materials and methods

### Extraction methods

One gram of dry biomass of *A. platensis* (dried in the shade at 50 °C, stored at room temperature; provided by Biomex, Jalisco, Mexico) was taken and suspended in different types of solvents (1 g/100 mL). The biomass pretreatment was done with different mechanical methods, and finally, phycocyanin extraction was determined after 1 h and 24 h of agitation. Biomass pretreatment was done with: (1) agitation (Jeiotech SK 71 Orbital/Reciprocal Shaker), (2) sonification (SB5200DTN Ultrasonic Cleaner, 40 kHz for 20 min), (3) sonotrode ultrasonication (VCX130 Vibra-Cell Ultrasonic Liquid Processor, intermittent sonication at 1 MHz for 5 min), and (4) freeze/thaw methods. The solvent used for the extraction process was water or phosphate buffer at pH 6 and 7. To finish the mechanical cell disruption, the biomass was separated by centrifugation at 8000 rpm for 15 min with a Thermo Scientific Multifuge X3R centrifuge at 4 °C.

### Determination of concentration and purity of phycocyanin

Phycocyanin concentration and purity were determined by spectrophotometry as described by Bennet and Bogard 1973 [44]. Concentration was calculated with the formula below using absorbance at 620 nm and 652 nm with a Biotek Eon™ spectrophotometer with a high-performance microplate. For purity, a ratio of A_620_/A_280_ was used.

Phycocyanin absorbs light at about 620 nm and emits fluorescence at about 640 nm [45]. The purity of C-PC is usually evaluated using an absorbance ratio of A_620_/A_280_, and a purity of 0.7 is considered as food grade [44], Absorbance at 620 nm indicates maximum C-PC absorption, while at 280 nm, it is due to the presence of other proteins in the solution [46]. C-PC purity is a ratio regarding the contamination of other proteins if present in the same sample.

$$\text{C}-\text{PC }\left(\frac{mg}{mL}\right) = \frac{({Abs}_{620}-0.474\left({Abs}_{652}\right))}{5.34}$$

$$\text{Yield }\left(\frac{mg}{g}\right) = (C-PC \times \text{Volume})/\text{Biomass}$$

### Partial purification of phycocyanin

Partial purification was carried out by the precipitation method with (NH_4_)_2_SO_4_, then centrifugation. The crude extract was subjected to direct precipitation with (NH_4_)_2_SO_4_ at 20%, 30%, 40%, 50%, 60%, and 70% and fractional precipitation (20–65%, 30–65%) using (NH_4_)_2_SO_4_, which was maintained overnight at 4 °C. The pellet was recovered by centrifugation at 4 °C and dissolved again in water for quantification and purity testing.

### Determination of phycocyanin stability at different storage conditions

Phycocyanin stability in the crude extract was evaluated with the following storage conditions in the dark: temperature: 4 °C, 25 °C, and − 20 °C and pH: 5, 6, and 7. The storage time was 5 weeks. The pH, purity, and concentration of phycocyanin were determined at 1 week intervals up to 5 consecutive weeks by spectrophotometer (Biotek Eon).

### Amino acid analysis

Replicate samples of *A. platensis* biomass after phycocyanin extraction were lyophilized and used for total amino acid analysis.

From the sample, 6 mg was weighed and a solution of 6 N hydrochloric acid was added and subsequently hydrolysis was started for 24 h in an oven at 100 °C. After hydrolysis, the sample was dried in the presence of nitrogen and dissolved with water and injected for gas chromatography (UPLC-QDa, Waters) for amino acid analysis along with the standards. For chromatographic analysis, an acetonitrile–water phase with formic acid and (heptafluorobutyric acid (HBFA) was used at a flow rate of 0.6 mL/min and an XBrige C18 column at a temperature of 40 °C. The protein was measured by the method currently recommended by the Food and Agricultural Organization (FAO): the sum of amino acids excluded tryptophan [47].

Essential amino acid index (EAAI) scores were calculated to show the protein quality still available in the biomass, as in Tabarsa et al. [48], using a human reference pattern established by the FAO/World Health Organization (FAO 1991) [49].

The EAAI was determined by the formula

$${\mathbf{EAAI}} \, = \, {\mathbf{10logEAA}}$$

where log EAA = 0.1[log(a_1_/a_1s_ × 100) + log(a_2_/a_2s_ × 100) + log(a_n_/a_ns_ × 100)] = 1.16; a_1_, …, a_n_ are the amino acid contents of *A. platensis* and a_1s_, …, a_ns_ are the average required levels of these amino acids in humans.

### Statistical analysis

All measurements were carried out in triplicate and Statgraphics Centurion XVI ver. 16.1.11 statistical software was used to develop unifactorial analysis of variance (ANOVA) by the Tukey LSD (least significant difference) method comparing treatments. If the statistical F-value showed significant differences, a multiple range test was applied to determine which treatment was different at a 95% confidence level.

## Results

### Phycocyanin yield from *A. Platensis* by standard extraction method

The extraction of phycocyanin was done with the method mentioned above, showing an intense blue color and a remarkable crude phycocyanin content of 0.286 g/g, which was 28.6% of the dry biomass with a purity of 0.46. It was found that the Mexican strain had a higher yield compared to previously reported strains of *A. platensis* [4].

Eriksen et al. [38] reported that high light intensity changes the characteristics of any photosynthetic system; on the other hand, too much light may cause photoinhibition. As a consequence, the pigment that absorbs the specific wavelengths of light becomes predominant [50].

Patel et al. [4] found that *Arthrospira* sp. had a higher content of phycobiliproteins (22.5% dry biomass) than *Phormidium sp.* (5.4% dry biomass.) and *Lyngbya* sp*.* (5.8% dry biomass). It was found that there was a maximum amount of C-PC in *Arthrospira* sp*.* of 17.5% of the dry biomass, compared to *Phormidium* sp. (4.1%) and *Lyngbya* sp*.* (3.9%), while allophycocyanin and phycoerythrin were present in lower amounts.

In our results, we found a higher amount of crude phycobiliproteins, which was 28.6% of the dry biomass of *A. platensis* and showed an intense blue color when extracted at a biomass/solvent ratio of 1:100.

### Optimized extractions of phycocyanin from *A. Platensis* by different methods

Several techniques have been reported to extract phycocyanin [51] from the biomass of *S. platensis* (dry, wet, and frozen). The cell walls of cyanobacteria are quite resistant [52]. They are formed of four layers: fibrils, peptidoglycans, proteins, and oligosaccharides, similar to gram-negative bacteria [53]. It has been reported that several methods rupture the cell wall, such as homogenization, sonication, microwave, supercritical fluid process, and disintegration of lysozyme [54–56]. The process must be efficient in terms of high extraction performance and must be respectful of the environment and further use for the food industry.

**Fig. 1:**
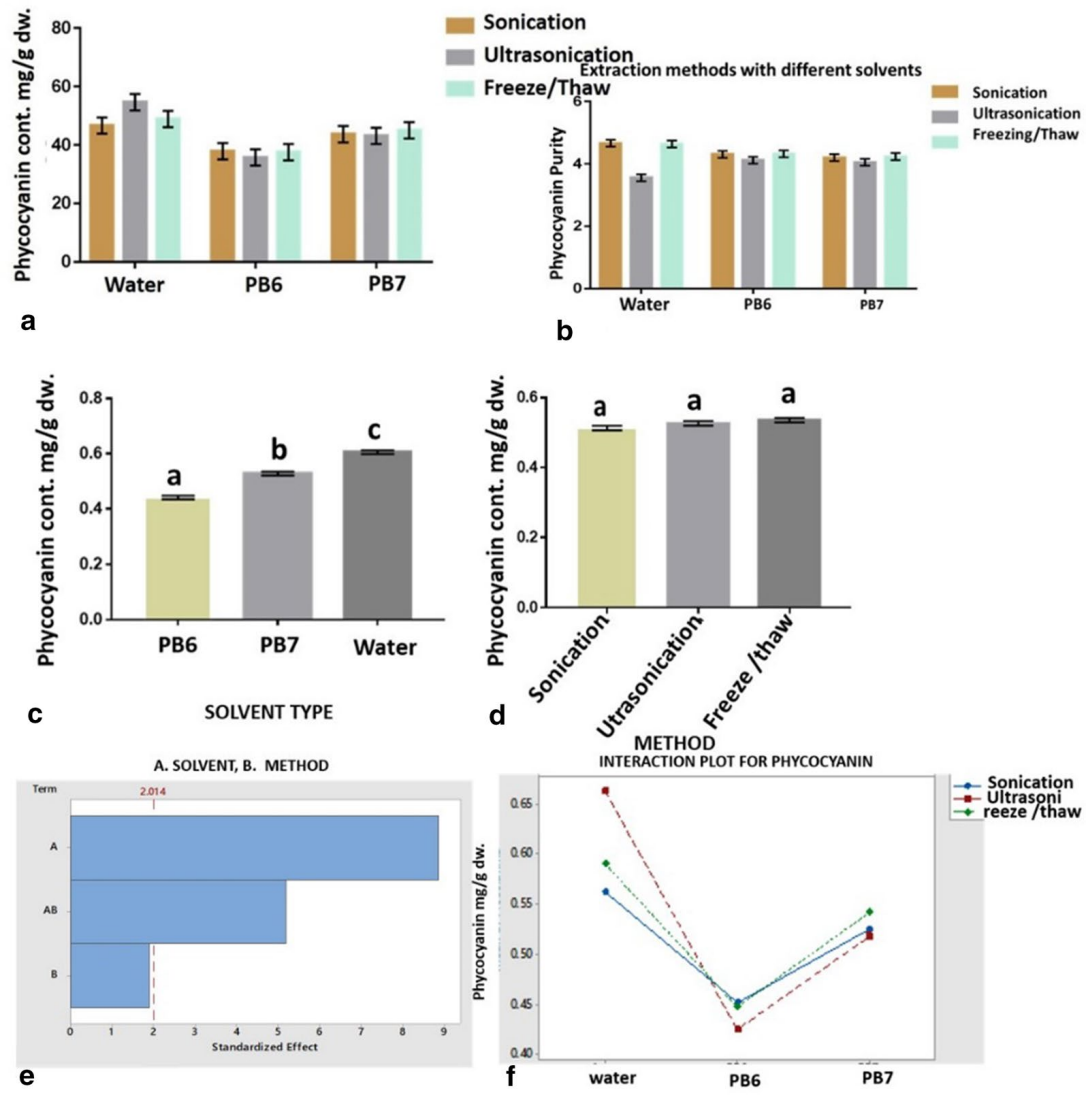
**a**, **b** Phycocyanin content and purity with different extraction methods and solvents from *A. platensis*. Interaction between solvent type and phycocyanin content (**c**, **e**). Interaction between extraction method and phycocyanin content. **d**, **f** Interaction between solvents, extraction methods, and phycocyanin content. Equal letters in the column shown that there is no significant difference between treatments; different letters have shown there exists a statistical difference (**c**, **d**)

We found higher phycocyanin content, 46.65–54.65 mg/g biomass, with water in all methods used compared to other solvents (Fig. 1). In the case of phosphate buffer at pH 6, phycocyanin content was 35.54–37.88 mg/g, and with phosphate buffer at pH 7, phycocyanin content was 43.13–45.02 mg/g.

Two solvents at two pH values and three types of mechanical pretreatment were used to break the cell walls gradually. The solvents that were used to evaluate the efficiency of phycocyanin extraction were water and phosphate buffer pH 6 and 7. Methods implemented were sonication, ultrasonication, and freeze/thaw. As observed by the Tukey test (Fig. 1a, b), the amount of phycocyanin extracted with water was statistically higher than with phosphate buffer. The statistical value *p* > 0.05 shows that there is a significant statistical difference among the solvents (Fig. 1c) but no significant statistical difference among the methods (Fig. 1d).

When we compare the same solvent between methods, ultrasonication showed the highest phycocyanin content, 54.65 mg/g with water, and the lowest was 35.54 with phosphate buffer at pH 6. It was observed that a higher amount of phycocyanin was extracted by ultrasonication, followed by freeze/thaw and sonication, as shown in Fig. 1c. Still, they were found statistically equal by the Tukey test (Fig. 1d). In the first graph of interaction (Fig. 1e), it was observed that the effect that most influenced our response was the solvent. Figure 1f shows that the highest amounts of phycocyanin were obtained with water and ultrasonication (54.65 mg/g).

### Phycocyanin purity analysis

The phycocyanin content with the freeze/thaw method was moderate, 48.88 mg/g, with a purity of 0.46, but the purity was lower with the ultrasonication method (0.35). The amount of phycocyanin extracted from the dry biomass with water as a solvent, using the ultrasonication method in the water bath (Fig. 1b), was 46.65 mg/g, with a purity of 0.46. The two extraction methods, sonication (in a water bath) and ultrasonication (electrode), can be recommended for industrial-scale production of phycocyanin since there was no statistical difference in the amount of phycocyanin content (Fig. 1d).

**Fig. 2:**
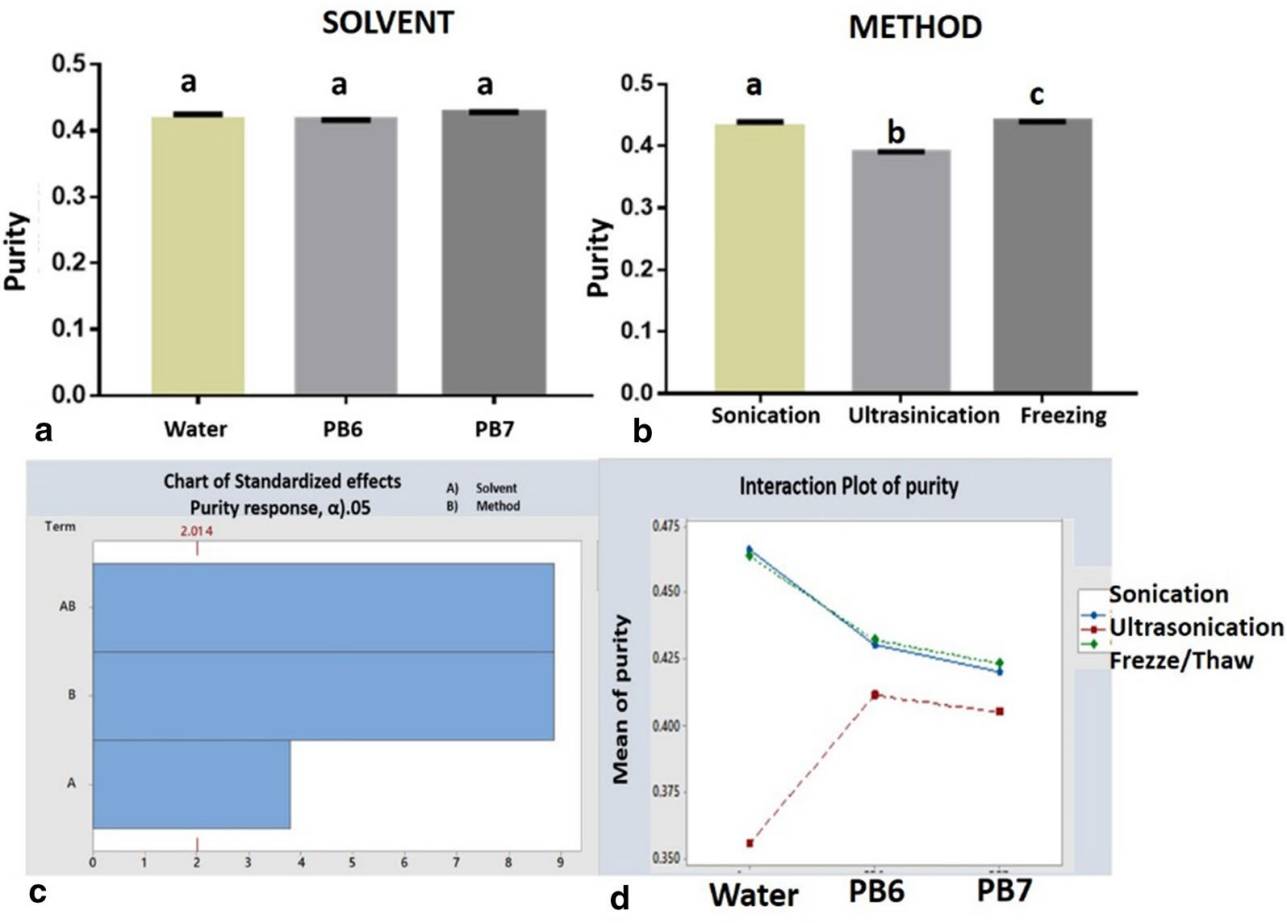
**a**–**d** Phycocyanin purity interaction with different extraction methods and solvents from *A. platensis*. Equal letters in the column shown that there is no significant difference between treatments; different letters have shown there exists a statistical difference (**a**, **b**)

However, the freeze/thaw method seems more attractive for large-scale extraction of phycocyanin without using much energy and with easy handling. The optimal conditions of the extraction methods for better purity were determined with the freeze/thaw method. In our work, we found that the extraction solvent (water) influenced the amount of phycocyanin, not the method (Fig. 1e, f). It would be advisable to use the sonication or freeze/thaw method since these obtained similar amounts of phycocyanin with higher purity (0.46). The statistical value *p* > 0.05 shows that there was no significant statistical difference among the solvents (Fig. 2a), but there was a significant statistical difference among the extraction methods, affecting purity (Fig. 2b).

In the interaction graphs (Fig. 2b, d), it was observed that the method of extraction influenced C-PC purity. It was found that with water, higher purity could be obtained with both sonication and freeze/thaw methods, which allowed phycocyanin extraction (Fig. 4d) with higher purity than the ultrasonication method.

## Phycocyanin stability at different storing temperatures and pH

### Stability analysis of phycocyanin at pH 5 at different temperatures

The temperature and pH of the solvent were evaluated for the crude extract of phycocyanin. These are the key factors that influence the stability of phycocyanin during storage. Starting with the same initial concentration at pH 5, after 3 weeks of storage at different temperatures, the phycocyanin content showed variation. After 5 weeks of storage, the phycocyanin content decreased to 15% at 25 °C, 83% at 4 °C, and 51% at − 20 °C. The most appropriate storage condition was 4 °C at pH 5. Citric acid used for acidification may act as a preservative of the phycocyanin solution to inhibit microbial growth.

### Stability analysis of phycocyanin at pH 6 at different temperatures

Phycocyanin starting at pH 6 was kept at different storage temperatures and evaluated at 1-week intervals. At room temperature (25 °C), the amount of C-PC was only 56.60%, stable from the initial amount after 5 weeks. At 4 °C, the stability was similar, 93.07% and 92.75% after the third and fifth weeks, respectively. At − 20 °C, the stability was found to be 95.24% and 94.32% after the third and fifth weeks, respectively. It was found that the most appropriate storage temperature at pH 6 was − 20 °C for better preservation of phycocyanin. The most considerable amount was conserved at 4 °C up to 35 days with 93% of C-PC (Fig. 5, Table 1); at − 20 °C the PC quantity was conserved up to 94% after 35 days, while at room temperature only 56% was preserved, an undesirable value. When compared with pH 5, the highest stability was found with pH 6 at − 20 °C.

**Table 1:** Phycocyanin stability (% of PC. content) dw. with different pH and storage temperature after 35 days

	pH-5	pH-6	pH-7
4 °C	83.04 ± 0.36	92.75 ± 0.69	73.09 ± 0.32
25 °C	14.55 ± 0.03	56.60 ± 0.17	25.64 ± 0.04
− 20 °C	51.03 ± 2.41	94.32 ± 0.05	98.69 ± 0.32

### Phycocyanin stability analysis (pH 7)

**Fig. 3:**
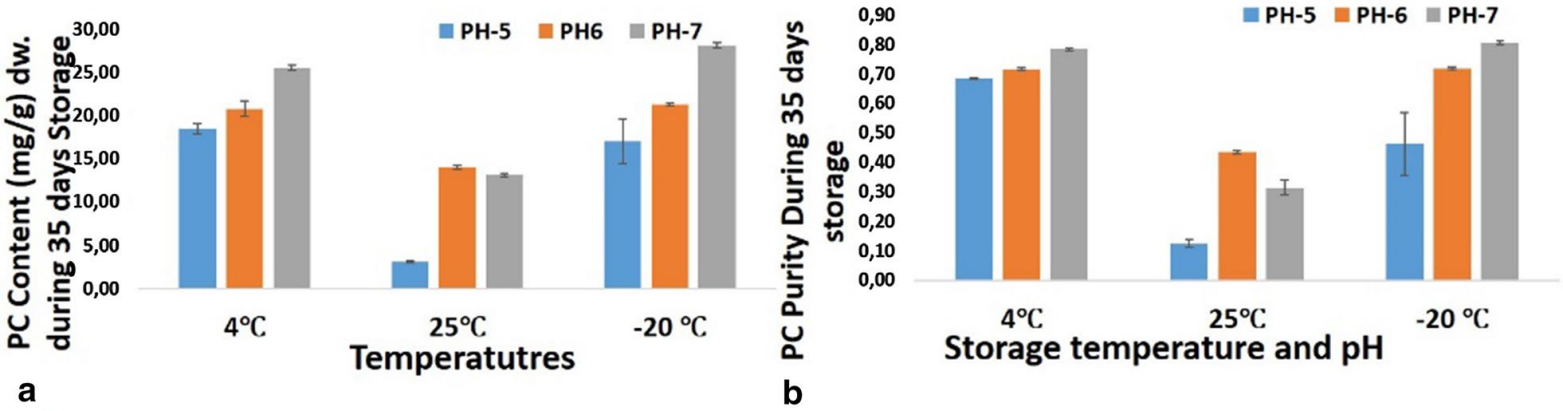
Phycocyanin content (mg/g) dw. (**a**) and purity (**b**) with different pH and storage temperature for 35 days. (dw: dry biomass weight)

Starting with pH 7 at room temperature (25 °C), only 25.64% of the initial amount of PC was found after the fifth week. At 4 °C, the stability was different, approximately 89.56% and 73.09% after the third and fifth weeks, respectively, at pH 7. At − 20 °C, the stability was approximately 98.77% and 98.69% after the third and fifth weeks, respectively (Fig. 3a, b, Table 1). We found that the most appropriate pH was 7, at − 20 °C. Potassium hydroxide was added to raise the pH of the phycocyanin solution to 7 from an initial pH of 6.4. The highest amount of C-PC was 89.5% at 4 °C up to 21 days. However, at − 20 °C, the amount conserved up to 21 days was 98.7%, while at room temperature, only 46% was retained. When stability at pH 5 and 6 was compared, the highest stability obtained was at pH 7 at − 20 °C.

### Interaction analysis of stability of PC with different pH, temperature, and time

At pH 5 the best stability was at 4 °C between the third and fifth weeks. At pH 6 the best stability was found at 4 °C and − 20 °C. The data obtained were similar. The best stability obtained was at − 20 °C and pH 7 between the third and fifth weeks. Among all pH values, it was observed that room temperature was not viable to account for phycocyanin stability, since the values obtained were very low compared to the other storage temperatures, 4 °C and − 20 °C. Between the two cold storage temperatures, at − 20 °C there was better stability at pH 6 and pH 7, but at pH 5 it was better at 4 °C. There was no significant difference in phycocyanin stability between the third and fifth weeks. It is also clear that pH 7 and − 20 °C was the best storage condition for phycocyanin stability, which was 98.7% for 35 days, although 40 °C was also good for phycocyanin stability at pH 6 and pH 7 (Fig. 3, Table 1). Stability was remarkable at − 20 °C up to 5 weeks, since there was very little loss of phycocyanin and it lasted longer (Fig. 3).

**Fig. 4:**
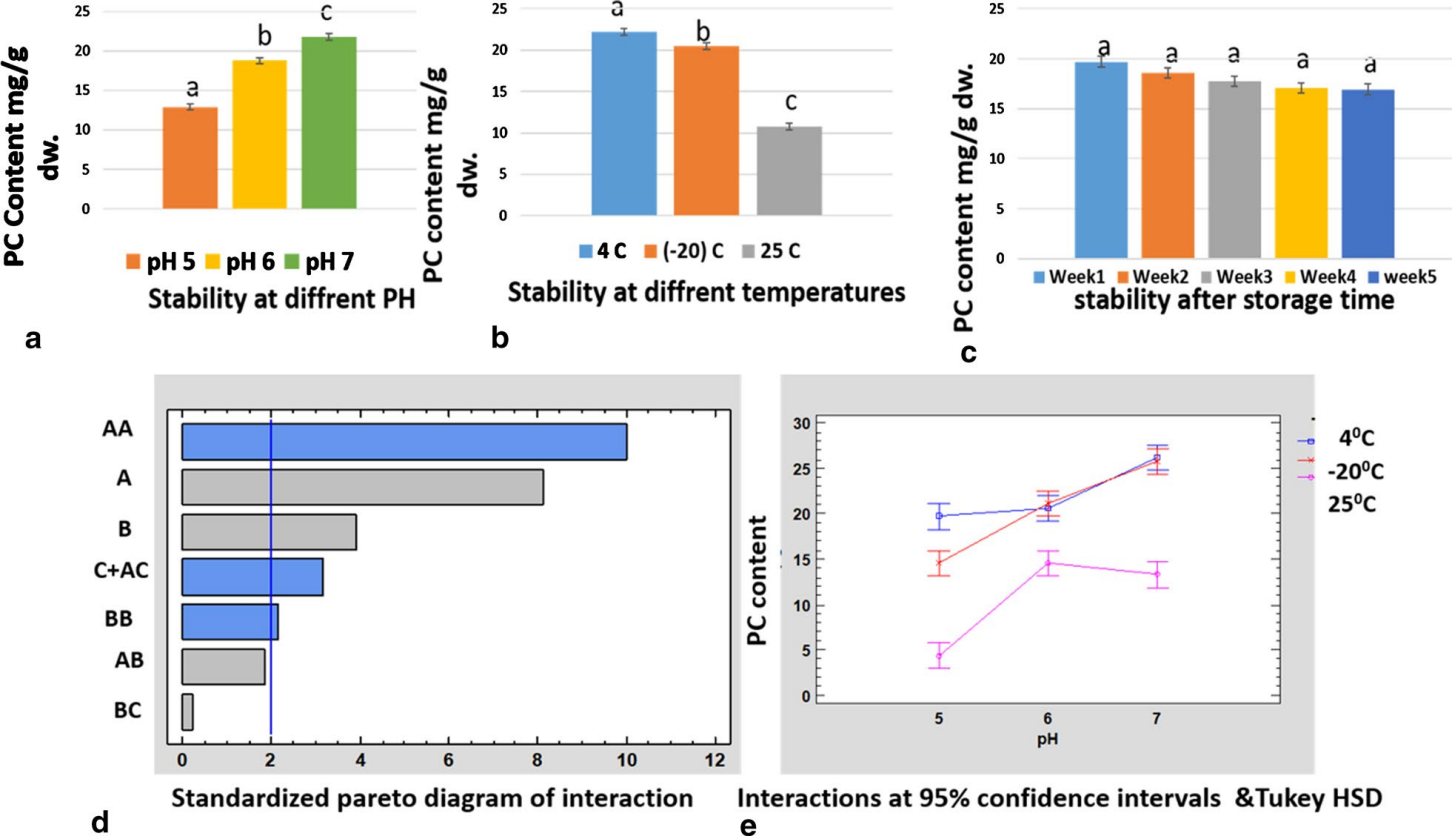
Interaction graph of phycocyanin stability with (**a**) pH (**b**) temperature, and **c** storage time (Tukey method and 95% confidence level); **d** standardized interaction; **e** interactions of Tukey HSD (honestly significant difference) (A: temperature; B: pH; C: storage time). Same letters in the column signify no significant difference between treatments; different letters signify statistical difference (**a**–**c**). PC, phycocyanin; dw, dry biomass weight

From the statistical analysis (ANOVA) and interaction graph (Fig. 4a–e), it was found that temperature had the most effect on stability, followed by pH and time. The statistical value *p* > 0.05 shows that there was no significant statistical difference in storage time regarding PC content (Figs. 4c) but there was a significant statistical difference in stability at different pH and temperature (Fig. 4a,b).

### Purity analysis of phycocyanin at different storage temperatures and pH

During storage conditions, the purity of phycocyanin may also vary. There are not many reports on the evaluation of purity during storage conditions. In our case, we found that at pH 5 the purity slowly decreased to 95% after 5 weeks at 4 °C, 30% after 5 weeks at 25 °C, and 74% after 5 weeks at − 20 °C. It was found that room temperature affected purity, to as low as 30%, and other cold temperatures did not have a significant effect on purity.

We found that at pH 6 purity remained stable with a slight decrease up to 98% after 5 weeks at 4 °C, 68% after 5 weeks at 25 °C, and 98% after 5 weeks at − 20 °C. This shows that normal temperature affects purity and cold temperatures have very little effect on purity at pH 6. At pH 7 the purity remained stable with a slow decrease up to 96% after 5 weeks at 4 °C, 46% after 5 weeks at 25 °C, and 99.7% after 5 weeks at − 20 °C. This shows that normal temperature affects purity, and cold temperatures have a minimal effect on purity at pH 7. It was also clear that pH 7 and − 20 °C was the best storage condition for phycocyanin, which was approximately 99.7% for 35 days.

**Fig. 5:**
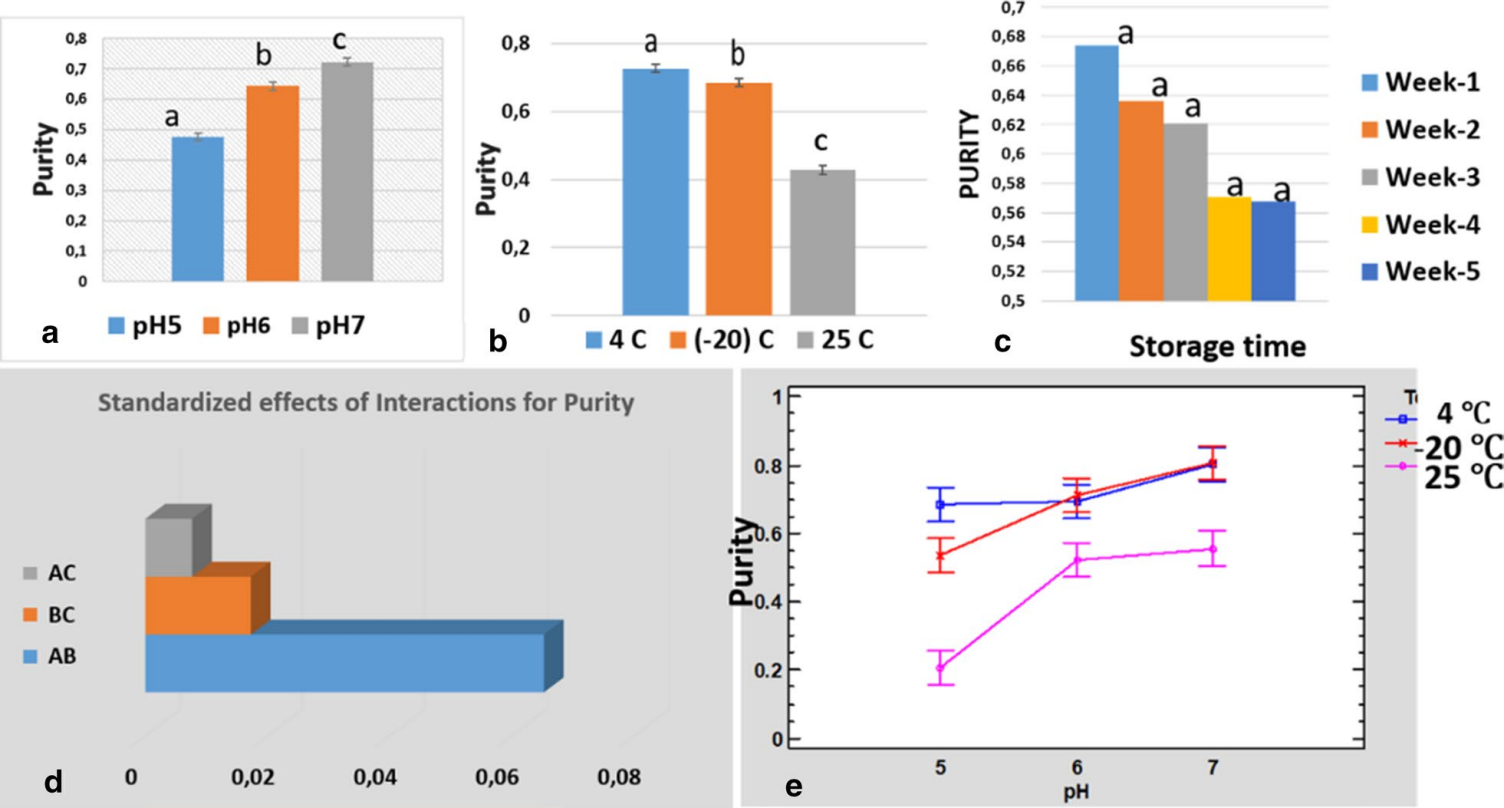
Interaction graph of phycocyanin purity with **a** temperatures, **b** pH and **c** storage time (Tukey method and 95% confidence level), **d** standardized **e** interaction Turkey HSD A = pH, B = Temperature and C = Storing time

From the statistical analysis (ANOVA) and interaction graph (Fig. 5d, e), it was found that pH had the most effect on purity, followed by temperature and time. The statistical value *p* > 0.05 shows that there was no significant statistical difference in storage time regarding C-PC purity (Fig. 5c) but there was a significant statistical difference in stability at different pH and temperature (Fig. 5a,b).

### Results of partial purification of phycocyanin

It was found that simple direct precipitation with 50%–70% of ammonium sulfate gave a purity of 0.39 to 0.82. However, when fractionation purification was used with 20–65%, 30–65%, and 40–65%, purity was 0.76–0.99. We found that the fractionation precipitation process was most effective when a fraction of 0–20% of the first precipitation was discarded, then the supernatant was precipitated up to 65%. The purity that was obtained was almost 1, which can serve better for food-grade applications (Table 2).

**Table 2:** Purity and quantity of phycocyanin during partial purification with ammonium sulfate precipitation

Direct precipitation	Purity	Quantity (mg/g)
20%	0.39 ± 0.04	4.53 ± 0.075
30%	0.59 ± 0.02	5.84 ± 0.49
40%	0.77 ± 0.00	5.74 ± 0.15
50%	0.77 ± 0.00	6.09 ± 0.13
60%	0.79 ± 0.00	7.00 ± 0.13
70%	0.82 ± 0.00	9.89 ± 0.26
0–20%/20–65%	0.99 ± 0.02	3.44 ± 0.13
0–30%/30–65%	0.81 ± 0.06	4.53 ± 0.16
0–40%/40–65%	0.76 ± 0.01	2.67 ± 0.099

Silva et al. found the best results with fractionation at saturations of 0–20% and 20–50%, which resulted in a product with a purity of 0.88. The results of purity by fractionation were better than those obtained by direct precipitation at a saturation of 50%.

### Biochemical composition of *A. Platensis*

**Fig. 6:**
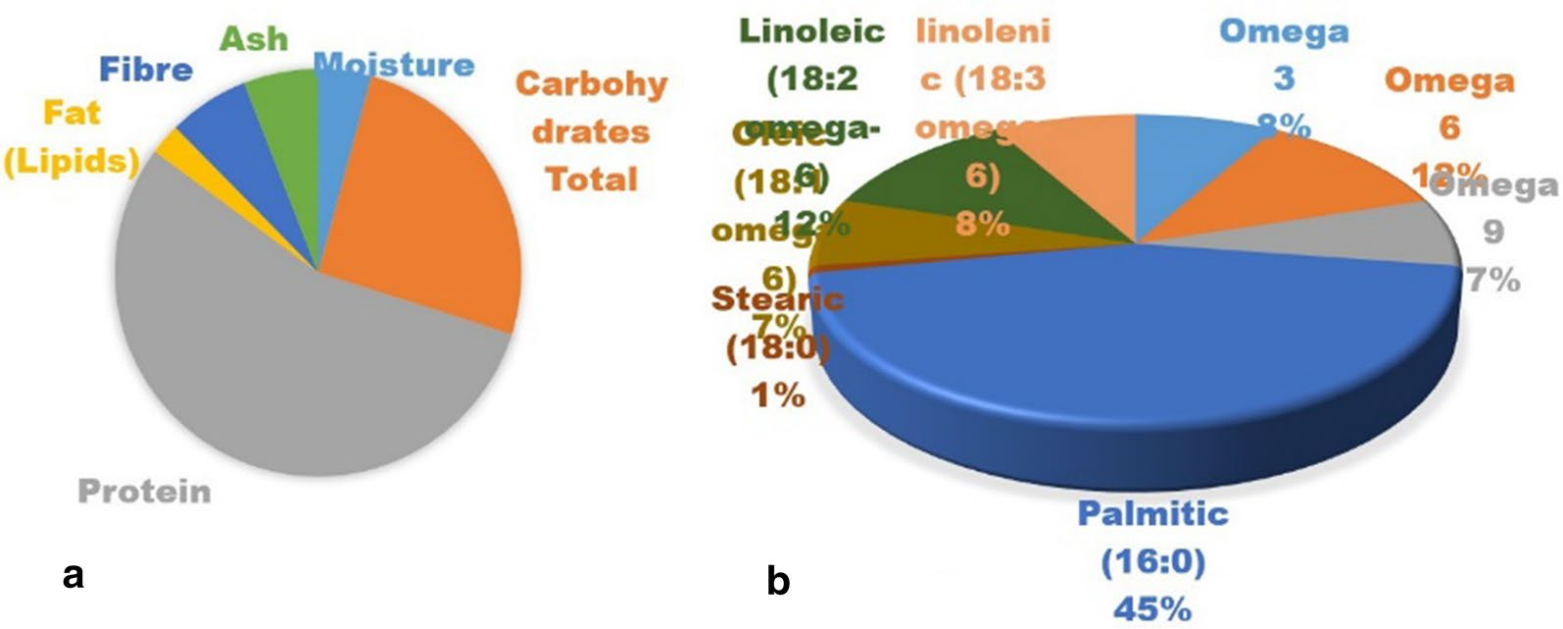
**a** Biochemical composition of *A. Platensis*, 6. **b** Fatty acid profile of *A. Platensis*

We analyzed the nutritional properties of *A. platensis* collected from Mexico, as they can vary according to climatic conditions or region of collection. This profile is important for the productivity of this microorganism regionally. In our *A. platensis* dry biomass powder, we got 60% protein and 28.5% carbohydrates (Fig. 6a). The fat percentage was low at 2.65%.

### Fatty acid profile of *A. Platensis*

We analyzed *A. platensis* to find the quality of fatty acids. We found 2.27% of total fatty acids of the dry biomass, of which 0.23% was omega-3 and 0.33% was omega-6. From the total fatty acids, 8% was omega-3 and 12% was omega-6. Palmitic acid was the most dominant fatty acid found, approximately 45% (Fig. 6b). This is a bit different from other reported species. The omega-6/omega-3 ratio in *A. platensis* species was found to be within the recommended range for human health benefits, approximately 1.43 [57].

### Amino acid profile of biomass after phycocyanin extraction

Microalgae are a rich source of protein, similar to traditional protein sources such as meats, eggs, soybeans, and milk [58]. Algae used for protein productivity have several benefits in terms of productivity and nutritional value. In this work, phycocyanin pigment was extracted to get a high-value product that can be used for natural coloring and as a functional ingredient. In the second step, we estimated the rest of the biomass, which was nearly 50%, that can be used as a protein source containing 33.38% of total protein (Table 3), which is a remarkable quantity in the same biomass. Its useful for animal and human food consumption, as the solvent used here was water, which is nontoxic for further use.

In our *A. platensis* species we found the presence of most of the essential amino acids (EAAs), including histidine (0.69%), isoleucine (24.56%), leucine (10.99%), lysine (1.84%), phenylalanine (6.45%), threonine (8.15%), and valine (7.84%). Only methionine and tryptophan were missing or not in the detectable range. Glutamic acid (12.92%) and proline (7.05%) contributed the major percentage of non-EEAs. The total percentage of EAAs was high in *A. platensis* species (20.21%), whereas non-EAAs was 12.17% of the biomass after phycocyanin extraction. The quality of protein was high due to the presence of a higher proportion of EAAs.

The amount of each amino acid was expressed as a percentage of total biomass after phycocyanin extraction. The essential amino acid index (EAAI) is a standard method for estimating the quality of a test protein [59]. In our protein quality evaluation, an EAAI score of 1.16 (> 0.95) demonstrated high-quality protein [60].

## Discussion

*Nostoc, Arthrospira*, and *Aphanizomenon* are protein-rich microalgae that have been used in the human diet for thousands of years [61]. In Mexico, Aztecs consumed a blue-green cake made from *Arthrospira species* [62]. However, there are no clear scientific reports showing the nutritional value of *Arthrospira* collected from Mexico. We found a notable amount of protein, 55% of the initial dry biomass, and a very good essential amino acid profile, 33%, after extraction of phycocyanin pigment from *A. platensis* collected from Mexico. Though the lipid amount was low, the fatty acid profile was quite interesting. Cyanobacteria may adapt to alter their light absorption characteristics to regulate photosynthesis according to the availability of light in different environments. So pigment content such as phycocyanin that is involved in light absorption may vary depending on the light intensity of the particular climatic conditions.

Various high-value compounds can be extracted from the biomass of different microalgae species; even the same bioactive compound varies within a species with different strains, environmental conditions, and culture conditions. More companies are focusing on commercializing bioactive compounds from microalgae using strategies related to the extraction and purification process, which accounts for 50–80% of production costs [63]. Hence it was important to explore efficient extraction methods for *A. platensis*, a native strain from Mexico (*A. platensis1tex* [37]) and to study stability conditions for further industrial use of the phycocyanin pigment.

The critical factor to consider during extraction is the proper control of pH, temperature, and ionic strength, which is crucial for the stability of the complex. Additionally, cell disruption is the critical factor for maximum extraction efficiency. In our work, physical methods were chosen for phycocyanin extraction (sonication, ultrasonication, and repeated freeze–thaw method), so as to not contaminate it with chemical residues, as it is intended to be used further in food. Some previous reports [64] suggested that extraction of phycocyanin with water did not differ much from phosphate buffer extraction (pH 7.4). Işıl et al. [65], recently reported a double extraction yield of phycocyanin from frozen biomass using distilled water compared to phosphate buffer. Some techniques to extract phycocyanin from *S. platensis* biomass in various physical forms (dry, wet, and frozen) have been reported [66]. The common methods reported for cell wall disruption are homogenization, sonication, microwave, supercritical fluid extraction, and lysozyme disintegration [63, 67, 68]. We combined solvents and methods to optimize the extraction process for dry biomass. The general separation process includes three stages: cell disruption, primary recovery, and purification. We used only easy mechanical methods for cell disruption, as methods such as enzymatic processes for cell disruption are expensive, and chemical methods can degrade, as proteins are very sensitive to it. Choosing the right cell disruption method is essential for better separation and recovery of the desired product.

Benavides and Rito-Palomares [69] found a 5.5 times higher protein release with sonication. Juneau and colleagues [70], found more unnecessary proteins from *P. cruentum* for B-phycoerythrin pigment by high-pressure cell disruption. Previous results showed that the phycocyanin recovery from freeze-dried biomass was approximately 100% and in sun-dried biomass was 95%, whereas in oven-dried biomass it was around 20% [69]. Our biomass was provided by a microalgae company (Biomax), and it was dried at 40–45 °C under shade conditions to minimize the loss of phycocyanin. We found a good amount of crude phycocyanin, approximately 28.6% of the dry biomass. It was quantified gravimetrically and showed an intense blue color when extracted with 1 g/100 mL. However, when quantified with a spectrophotometer, we found that phycocyanin content was 4.88% of the biomass. That may be due to pure phycocyanin quantification by the spectrophotometry method. Still, the phycocyanin yield was higher than other reported species. With our work, this species shows potential to be used by the industrial sector for phycocyanin production.

Phycocyanin is very sensitive to heat and undergoes drastic changes when exposed to high temperatures [35]. Hence, in our study, we only used room temperature and low temperatures. There are other reports regarding the analysis of thermal degradation of phycocyanin in solution with the addition of stabilizers or preservatives [71]. However, the methodology used was the chemical kinetics of degradation. We used only natural conditions without adding other stabilizers. Still, for handling at certain pH, we added citric acid (0.1%) and KOH (5 M), but the minimal amount (50–100 uL) does not deviate much about the volume and concentration of phycocyanin. The aqueous solutions of phycocyanin were subjected to different storage temperatures to evaluate the effect of pH, time, and temperature. The condition in which the pigment showed stability implies that it retains the properties that can make it valuable in the food and cosmetic industries in the future. When we compared two cold temperatures, it showed similar purity results for 35 days, suggesting that phycocyanin can be used in cold-storage foods as a functional ingredient. Apart from that, ascorbic acid aggregation was used in a very low quantity (0.1%) to manipulate pH conditions, and served as a preservative, without causing any toxicity. Regarding stability, our product also showed similar results as previous studies. It was only stable at cold temperatures of 4 °C to − 20 °C up to 4–5 weeks and suitable pH of 6–7.

Phycocyanin purification from crude algae extracts is usually carried out by a combination of methods such as ammonium sulfate precipitation, ion-exchange chromatography, and gel filtration chromatography. The purification steps affect 50–90% of the productivity costs [63] and can be enhanced approximately two- to threefold from the purity ratio of the crude extract [4, 72]. Our intention in purification was to obtain a natural food colorant for food-grade application, and we got 0.99 purity by 0–20% and 20–65% with the ammonium sulfate precipitation method. Hence we avoided several purification steps, to not make the process expensive. Ammonium sulfate is considered a safe food additive by the US Food and Drug Administration [68], and in the European Union. It is used as an acidity regulator in flours and breads [73]. Fractionation makes the purification more interesting for industry, as this is a key factor to be taken into account, considering the costs of the process. It is extremely advantageous to obtain the maximum yield and purity of the byproduct in each step. Therefore, optimization could provide a byproduct with the required degree of purity with fewer purification steps. There are several other methods, such as HPLC and chromatographic methods [74], for purification up to 85% of C-phycocyanin and allophycocyanin [75]. However, the yield of the desired product is decreased significantly, hence the cost increases. For food-grade applications, the level of purity of phycocyanin of 0.7 is sufficient to include it as a functional food ingredient.

The high protein content of algae means that they are gaining popularity for use as animal feed, including aquaculture, farm animals, and pets. An estimated 30% of global algal production is intended to be used as animal feed, and Arthrospira biomass with more than 50% protein is mainly used as a feed supplement [76, 77]. Arthrospira proteins are almost complete with most essential amino acids present, forming 47% of total protein weight [78]. Arthrospira cells have a relatively fragile envelope of murein [79], showing very high digestibility of the proteins (83–90% of dry biomass) [80, 81].

Essential fatty acids (especially omega-3 and omega-6) are important for human health in reducing the risk of cardiovascular diseases, controlling levels of blood cholesterol, maximizing brain function, improving visual acuity, reducing inflammation, and alleviating arthritis [82]. Therefore, the content of these fatty acids is critical for microalgal use. The human requirement for essential fatty acids is within 1–2% [83, 84] as they affect the immune system [85]. The recommended ratio of omega-6/omega-3 is between 0.25 and 1 for humans [57]. In cases of essential fatty acid deficiency, Arthrospira has always been recommended as a nutritional supplement [86]. *S. maxima* contains 1–2% gamma-linolenic acid of the dry matter [87], compared to *S. platensis*, in which it is 4% of the dry weight. Thus Arthrospira can be considered one of the best known sources of gamma-linolenic acid [88].

Additionally, the amino acid profile was estimated to find out the quality of protein. The quality of protein can vary dramatically, depending on the source and types of essential amino acids present [50]. Animal sources of protein are generally rich sources of EAAs, whereas plant proteins often lack one or more essential amino acids [89].

## Conclusions

The most relevant conclusion obtained in this work is that the *A. platensis* strain showed excellent yield of crude phycocyanin, which was higher than previous reports: 29% of dry biomass with a purity level of 0.46. The efficient extraction process is a green process with water and without any solvent contamination, making it a less expensive method for further food-grade applications. The parameter that most influences the stability of C-phycocyanin is temperature. At a pH range between 6 and 7, phycocyanin presents greater stability, with a maximum value of 73–98% of quantity and 96–99% of purity over 35 days. At low temperatures (− 20 °C and 4 °C) phycocyanin is more stable for at least 35 days.

## Data Availability

The datasets generated and/or analysed during the current study are not publicly available, but are available from the corresponding author on reasonable request.

## Abbreviations

C-PC: Phycocyanin
EAAI: Essential amino acid index
FAO: Food and Agriculture Organization
ANOVA: Analysis of variance
HSD: Honestly significant difference
